# Heterologous expression of rice *9*-*cis*-*epoxycarotenoid dioxygenase 4* (*OsNCED4*) in *Arabidopsis* confers sugar oversensitivity and drought tolerance

**DOI:** 10.1186/s40529-018-0219-9

**Published:** 2018-01-15

**Authors:** San-Gwang Hwang, Chia-Yun Lee, Ching-Shan Tseng

**Affiliations:** 1 Department of Horticulture, National Chung Hsing University, 145 Xingda Road, South District, Taichung, 40227 Taiwan, ROC; 2 Institute of Plant and Microbial Biology, Academia Sinica, Taipei, 11529 Taiwan, ROC; 3 Taiwan Agricultural Research Institute, Council of Agriculture, Executive Yuan, No.189, Zhongzheng Road, Wufeng District, Taichung, 41362 Taiwan, ROC

**Keywords:** Abscisic acid, 9-*cis*-epoxycarotenoid dioxygenase, Seed germination, Post-germination growth arrest, Drought tolerance

## Abstract

**Background:**

The *9*-*cis*-*epoxycarotenoid dioxygenases OsNCED4* was cloned from rice in conjunction with *OsNCED 1*-*3* and *5*, of which *3* has been shown to function in ABA biosynthesis and alteration of leaf morphology. In higher plants, NCEDs have been shown to be key enzymes controlling ABA biosynthesis and belong to a differentially expressed gene family. Aside from *OsNCED3*, it remains largely unknown if other *OsNCED* genes are involved in ABA biosynthesis in rice. Thus, transgenic Arabidopsis plants overexpressing *OsNCED4* were generated in the 129B08/*nced3* mutant background to explore *OsNCED4* function in ABA biosynthesis.

**Results:**

Heterologous expression of *OsNCED4* in Arabidopsis increased ABA levels and altered plant size and leaf shape, delayed seed germination, caused sugar oversensitivity in post-germination growth, and enhanced tolerance to drought. The native *OsNCED3* and *OsNCED4* promoters were expressed in an overlapping pattern in rice seeds and young seedlings, suggesting possible functional redundancy between *OsNCED3* and *OsNCED4*. At the one-leaf stage, similar regulation of *OsNCED3* and *OsNCED4* gene expression in roots or leaves in response to moderate salt stress (150 mM NaCl) was observed.

**Conclusion:**

Like *OsNCED3*, *OsNCED4* is functionally active in ABA biosynthesis in rice. *OsNCED3* and *OsNCED4* might play redundant roles in controlling ABA biosynthesis in rice, as suggested by GUS staining assay, but this should be further analyzed through complementation of rice *NCED* knockout mutants.

**Electronic supplementary material:**

The online version of this article (10.1186/s40529-018-0219-9) contains supplementary material, which is available to authorized users.

## Background

The functions of abscisic acid (ABA) in regulation of seed dormancy, seedling growth and development, stomatal closure, and stress tolerance is well studied (Jiang and Yu 2009; Zhu et al. 2009, 2011; Waterland et al. 2010; Gao et al. 2011; Bauer et al. 2013; Merilo et al. 2015). Connections among ABA, molecular signaling components, and nutrition have been identified. For example, Jiang and Yu (2009) suggested that the transcription factor AtWRKY2 regulates seed germination and post-germination developmental arrest in Arabidopsis via its response to ABA. At the same year, Zhu et al. (2009) reported that the glucose-induced germination delay in rice seeds is due to the prevention of ABA degradation, rather than an increase in ABA biosynthesis. Zhu et al. (2011) further elaborated that downregulation of *CYP707A2* expression, a gene encoding an ABA 8′-hydroxylase involved in ABA catabolism, and the subsequent reduction in ABA degradation, are closely associated with the delay of seed germination and seedling growth in Arabidopsis. More recently, Gao et al. (2011) characterized the protein function of AtCPR5 and revealed that seed germination and early seedling growth are independently regulated through the ABA and lipoxygenase (LOX) pathways.

ABA regulates numerous physiological responses in addition to seed germination and seedling growth. Previous reports have shown that ABA synthesis in guard cells is essential and sufficient for stomatal closure in response to declined relative humidity (Bauer et al. 2013; Merilo et al. 2015). Waterland et al. (2010) demonstrated the involvement of ABA in drought tolerance. The roles of ABA and ABA signaling in plant abiotic stress responses, including drought tolerance, have been recently reviewed and discussed (Sah et al. 2016; Vishwakarma et al. 2017).

In higher plants, 9-*cis*-epoxycarotenoid dioxygenases (NCEDs) are thought to be the key enzymes controlling ABA biosynthesis and stress tolerance (Iuchi et al. 2001; Sun et al. 2012; Vishwakarma et al. 2017). To date, five rice *NCED* genes have been reported (Oliver et al. 2007; Welsch et al. 2008; Zhu et al. 2009). However, only *OsNCED3* (GenBank Accession No. AY838899) was characterized as functionally active in ABA biosynthesis (Hwang et al. 2010). The biological functions of the other four rice *NCED* genes, *OsNCED1* (GenBank Accession No. AY838897), *OsNCED2* (GenBank Accession No. AY838898), *OsNCED4* (GenBank Accession No. AY838900) and *OsNCED5* (GenBank Accession No. AY838901) have yet to be deciphered. It remains unknown whether rice *OsNCED4* gene is involved in ABA biosynthesis or ABA-regulated physiological processes.

Previous studies of the *NCED* gene family in Arabidopsis revealed that different *NCED* genes function in different plant tissues to regulate ABA biosynthesis (Iuchi et al. 2001; Tan et al. 2003; Lefebvre et al. 2006; Agustí et al. 2007; Martínez-Andújar et al. 2011). For example, *AtNCED3* is primarily induced at high levels in leaves in response to water stress (Endo et al. 2008), and *AtNCED6* and *AtNCED9* appear to be major players regulating ABA biosynthesis in developing seeds (Lefebvre et al. 2006). More recently, Huo et al. (2013) found that lettuce *LsNCED4* is required for heat-inhibition of seed germination and its expression in leaves is induced by heat but not water stress. In contrast, *LsNCED2* and *LsNCED3* are both induced by water stress but not heat.

A real-time RT-PCR analysis of *OsNCED3*, *OsNCED4*, and *OsNCED5* in roots of rice seedlings subjected to salt or ABA treatment revealed that these three *NCED* genes are salt- and ABA-inducible (Welsch et al. 2008). In transgenic rice plants harboring an *OsNCED3*::*gfp* transgene, the *OsNCED3* promoter activity is strongly increased in the roots and leaves under drought and high-salt (400 mM NaCl) conditions, but little to no activity can be observed in grains and flowers (Bang et al. 2013). It remains unclear if *OsNCED3* and *OsNCED4* are expressed in an overlapping or non-overlapping pattern in rice seeds and leaves.

In the present study, the rice *OsNCED4* gene was heterologously expressed in the Arabidopsis 129B08/*nced3* mutant to test if *OsNCED4* may complement the 129B08/*nced3* mutant phenotype. We used the Arabidopsis 129B08/*nced3* mutant rather than rice *OsNCED* mutant because no rice *OsNCED* mutant was available at the time when we initiated this study. The 129B08/*nced3* mutant was a T-DNA insertion mutant requested from the Nottingham Arabidopsis Stock Center. The *OsNCED4* overexpression line was used to characterize *OsNCED4* function in ABA biosynthesis, seed germination, post germination growth, and drought tolerance.

## Methods

### Plant materials and growth conditions

The Arabidopsis wild type and mutant plants used in this study were in the Columbia (Col-0) background. Arabidopsis plants overexpressing the rice *OsNCED4* transgene in the 129B08/*nced3* mutant background were denoted as N4C (for complementation). The growing conditions were described previously (Hwang et al. 2010). Cold-pretreated seeds (4 °C, 4 days) from WT, 129B08/*nced3*, and the transgenic lines N4C-1, and N4C-2 were grown either on water agar medium made up by adding 0.7% Phytoagar (Duchefa Biochemie, Haarlem, The Netherlands) in autoclaved ddH_2_O, on agar plates supplemented with 0.2 or 4% glucose and half-strength MS, or on soil for the period of time specified below.

### Transgenic plant isolation

Rice *OsNCED4* gene cloning, plasmid construction and transformation, and screening of homozygous transgenic lines were performed using the procedures described previously (Hwang et al. 2010), except that *OsNCED4* was overexpressed instead of *OsNCED3* and the binary vector used in this study was modified from pCAMBIA1300 rather than pCAMBIA1281Z. The modified pCAMBIA1300 vector used in this study contained a 35S promoter. Two lines, N4C-1 and N4C-2, were subsequently subjected to phenotypic comparison, germination test, ABA assay, analysis of post-germination growth arrest, and determination of drought tolerance.

To test promoter activity in rice, the *OsNCED3* promoter, encompassing 2407 bp upstream of the ATG start codon of *OsNCED3*, and the *OsNCED4* promoter, a total of 1939 bp upstream of the ATG start codon of *OsNCED4*, were amplified by polymerase chain reaction (PCR) and fused to a β-glucuronidase (GUS) coding region in the pCAMBIA1305.1 binary vector. The binary vector was then used to introduce the *OsNCED3*::*GUS* or *OsNCED4*::*GUS* constructs into Japonica rice variety TNG67. Homozygous transgenic rice plants were screened by using 70 mg/L hygromycin B (InvivoGen, USA). Two independent homozygous transgenic lines carrying the *OsNCED3*::*GUS* construct (denoted N3P-1 and N3P-2) and two independent homozygous transgenic lines carrying *OsNCED4*::*GUS* (denoted N4P-1 and N4P-2) were randomly chosen for further study.

### Reverse-transcription polymerase chain reaction (RT-PCR)

In Arabidopsis, the procedures for total RNA extraction, RNA reverse transcription, and polymerase chain reaction, and the sequences of the *AtUBQ5* and *AtNCED3* gene-specific primers were previously described in Hwang et al. (2010). Other gene-specific primers used in this study were for *OsNCED4*: forward primer 5′-CCGTCCAAGGTGAAGGTGGC-3′, and reverse primer 5′-CTTCTCCGCCGTGCCGCTC-3′.

### Germination test

Three independent batches of cold-pretreated seeds from WT, 129B08/*nced3*, N4C-1, and N4C-2 were grown on water agar medium. The total number of germinated seeds was counted daily for a period of 7 days. In another set of experiments, seeds from the above-mentioned plants were grown on agar plates supplemented with 0.2 or 4% glucose (Glc) and half-strength MS for 7 days. Seeds were considered germinated when the radical protruded at least 2 mm from the seed coat.

### Phenotypic comparisons

Cold-treated seeds from WT, 129B08/*nced3*, N4C-1, and N4C-2 were grown in soil for 35 days and then subjected to phenotypic comparisons. To compare leaf shape, the fifth leaves were sampled after 35 days for width:length ratio determinations.

### ABA assay

Seedlings grown on agar plates supplemented with 0.2 or 4% glucose for 10 days were harvested for ABA analysis. The ABA assay followed the methods described in Lin et al. (2007). Briefly, ABA was first extracted from the plant samples, the extraction supernatants were then dried in a SpeedVac concentrator (miVac Duo concentrator, Genevac Ltd, Ipswich, UK), resuspended, and purified by filtering through a polyvinylpolypyrrolidone column and C18 cartridges (Hsu and Kao 2003). ABA content was determined by using Agdia Phytodetek^®^ ELISA kits according to the manufacturers’ instructions.

### Expression analysis of ABA-inducible genes by quantitative real-time PCR

To further confirm the involvement of *OsNCED4* in ABA biosynthesis, expression analyses of ABA-regulated genes, such as *AtKIN2* (At5g15970) and *AtRD29A* (At5g52310) (Zimmerli et al. 2008), were performed in WT, 129B08/*nced3*, N4C-1, and N4C-2 by quantitative real-time PCR (qRT-PCR). Seeds from the above-mentioned genotypes were grown on agar plates supplemented with 0.2 or 4% glucose for 10 days. The seedlings were then subjected to total RNA extraction using the RNeasy Plant Mini Kit (Qiagen, Germany). The qRT-PCR assay was followed the procedures described in Chen et al. (2014).

### Relative water loss tests

To determine relative water loss, the rosette leaves of 3-week-old soil grown WT, 129B08/*nced3*, N4C-1, N4C-2 plants were excised, put on plastic weigh boats and stored in an electronic dry box (Model-D-60C, EDRY Co., Ltd, Taichung, Taiwan, ROC). The relative humidity inside the electronic dry box was around 60%. The fresh weights of the rosette leaves were recorded every 30 min for a period of 3 h.

### Drought treatment

For drought treatment, 9 cm × 7 cm (diameter × height) plastic pots containing 53-day-old, well-watered WT, 129B08/*nced3*, or N4C (N4C-1 and N4C-2) lines were at first watered to field capacity by soaking the pots in a plastic tray containing water until the soil was saturated and removing the excess water in another plastic tray. Water was then withheld for 9 and 13 days to evaluate the drought resistance among the genotypes. The degree of drought resistance was determined by visual symptoms of foliage wilt.

### Rice genomic DNA extraction and polymerase chain reaction (PCR)

Seeds derived from TNG67, N3P-1, N3P-2, N4P-1, and N4P-2 rice plants were germinated and grown in half-strength Kimura B solution (Hsu and Kao 2008) for 1 week. Genomic DNA was then extracted from these 1-week-old seedlings using a Plant Genomic DNA Purification Kit (GeneMark, Taiwan). The primer pairs N3P-F (5′-CTGTCAACTTCAAGCTTGGG-3′) and GUS-R (5′-GCACGATACGCTGATCCTTC-3′) or N4P-F (5′-GCAGTGGTATTGTGACAGAC-3′) and GUS-R were used in the PCR reaction to verify the integration of the *OsNCED3*::*GUS* or *OsNCED4*::*GUS* constructs in N3P or N4P transgenic rice plants, respectively.

### GUS staining of transgenic rice plants

Rice husks harvested at heading stage, dehusked seeds, and dehusked seeds imbibed in water for 24 h at 25 °C from the four transgenic lines (N3P-1, N3P-2, N4P-1, and N4P-2) were subjected to GUS staining according to procedures described by Jefferson et al. (1987). One-week-old seedlings of N3P-1, N3P-2, N4P-1, and N4P-2 grown in half-strength Kimura B solution supplemented with or without 150 mM NaCl were sampled for GUS histochemical staining following the same procedures.

## Results

In this study, reverse genetic approaches were used to determine if rice *OsNCED4* is functionally active in ABA biosynthesis. GUS staining of transgenic rice plants harboring an *OsNCED3*::*GUS* or *OsNCED4*::*GUS* construct was carried out to determine the expression patterns of these two genes in developing seeds and seedlings under salt stress.

### Transgenic Arabidopsis plants overexpressing *OsNCED4*

Comparison of the amino acid sequence between OsNCED3 and OsNCED4 indicated that OsNCED3 and OsNCED4 share approximately 79% sequence homology and 71% sequence identity. Similar to Arabidopsis NCED3, both OsNCED3 and OsNCED4 proteins contain a putative plastid-targeting peptide near the N-terminal end and four conserved histidine residues essential for catalytic acticity (Additional file 1). Our previous study indicated that *OsNCED3* plays a role in ABA biosynthesis (Hwang et al. 2010). To verify whether *OsNCED4* may play a role in ABA biosynthesis, we generated Arabidopsis transgenic plants overexpressing *OsNCED4*.

**Fig. 1:**
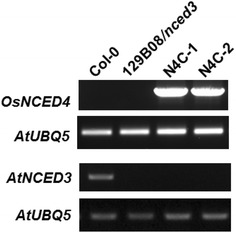
RT-PCR analysis of native *AtNCED3* and transgenic *OsNCED4* expression in Arabidopsis plants; Col-0, 129B08/*nced3* mutant, and two *OsNCED4*-overexpressing (N4C) transgenic lines

Reverse transcription (RT)-PCR was used to confirm the presence of the *OsNCED4* transcript in homozygous transgenic Arabidopsis plants. Our results indicated that *OsNCED4* transcripts were present in the two N4C transgenic lines, but not in Col-0 or 129B08/*nced3* mutant plants. *AtNCED3* transcripts were detected only in Col-0 (Fig. 1). These results confirmed the successful transformation and transcription of the *OsNCED4* transgene in the 129B08/*nced3* mutant background in two N4C transgenic lines. The 129B08/*nced3* mutant line maintained the lack of a functional *AtNCED3* gene.

### Analysis of leaf morphology and seed germination in *OsNCED4*-*overexpressing Arabidopsis*

**Fig. 2:**
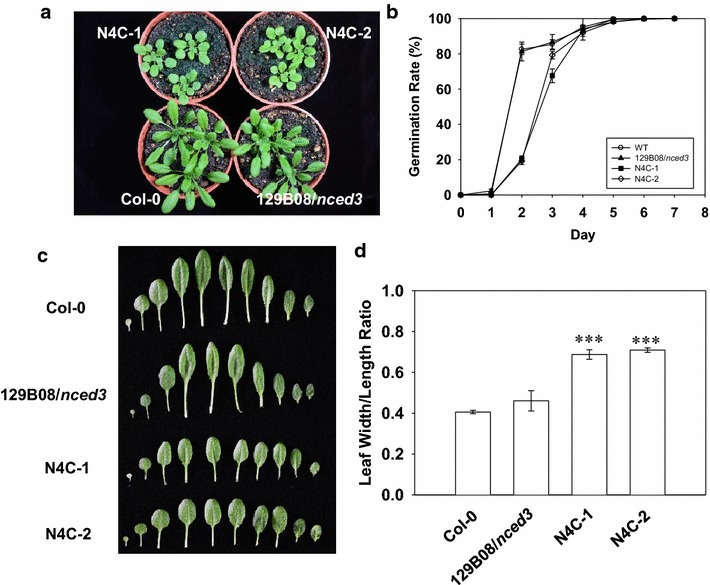
Phenotypic comparison of 35-day-old, soil-grown Col-0, 129B08/*nced3* mutant, and two *OsNCED4*-overexpressing transgenic lines. **a** Above-ground plant phenotype. **b** Germination rate, calculated from 100 seeds for each line, with mean ± SD of three independent experiments. **c** Leaf shape. **d** Width-to-length ratio of the fifth leaf. In **d**, the values are the mean ± SD of three independent experiments, each with nine plants. ***P < 0.001, Student’s *t* test

Heterologous expression of *OsNCED4* in the 129B08/*nced3* mutant reduced plant size (Fig. 2a), delayed seed germination (Fig. 2b), caused smaller and rounder rosette leaves (Fig. 2c), and increased the leaf width-to-length ratio to a value significantly closer to one, indicating that the leaf shape was rounder in the two N4C transgenic lines (Fig. 2d).

**Fig. 3:**
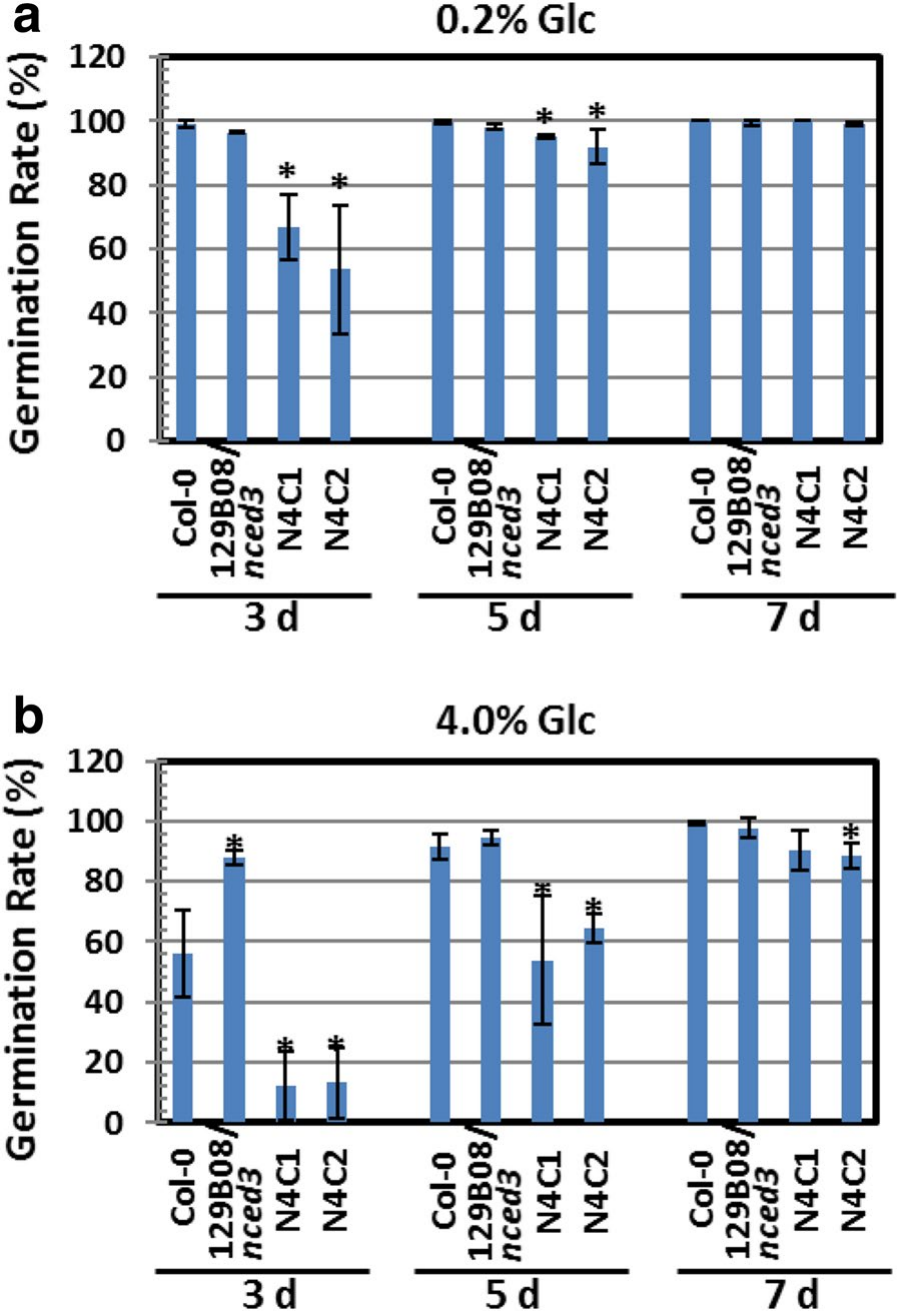
Heterologous expression of *OsNCED4* delayed seed germination in Arabidopsis. **a** Germination rate of seeds from Col-0, 129B08/*nced3* mutant, and two *OsNCED4*-overexpressing transgenic lines grown on agar medium supplemented with 0.2% Glc for 3, 5, and 7 days. **b** Germination rate of the same transgenic lines grown on agar medium supplemented with 4% Glc for 3, 5, and 7 days. The values are the mean ± SD of three independent experiments, each with 80–100 seeds. *P < 0.05, Student’s *t* test

Overexpression of *OsNCED4* shifted the bulk of the germination by delay 1–2 days compared to wild type and its mutant background. Germination did reach 100% by day 6 which was similar to wild type (Fig. 2b). While the germination rate of the 129B08/*nced3* mutant was similar to that of Col-0 in media either lacking Glc or containing low amounts of Glc (0.2% Glc) (Figs. 2b, 3a), the rate in this mutant was higher than that of Col-0 under high Glc (4.0% Glc) conditions 3 days after sowing (Fig. 3b). On the other hand, the transgenic lines overexpressing *OsNCED4*, N4C-1 and N4C-2, showed a seed germination delay at all levels of Glc in the media, from that lacking Glc (Fig. 2b) to those containing both low and high Glc levels (Fig. 3a, b).

### Heterologous expression of *OsNCED4* in the 129B08/*nced3* mutant affected post-germination growth and ABA content under high glucose conditions

**Fig. 4:**
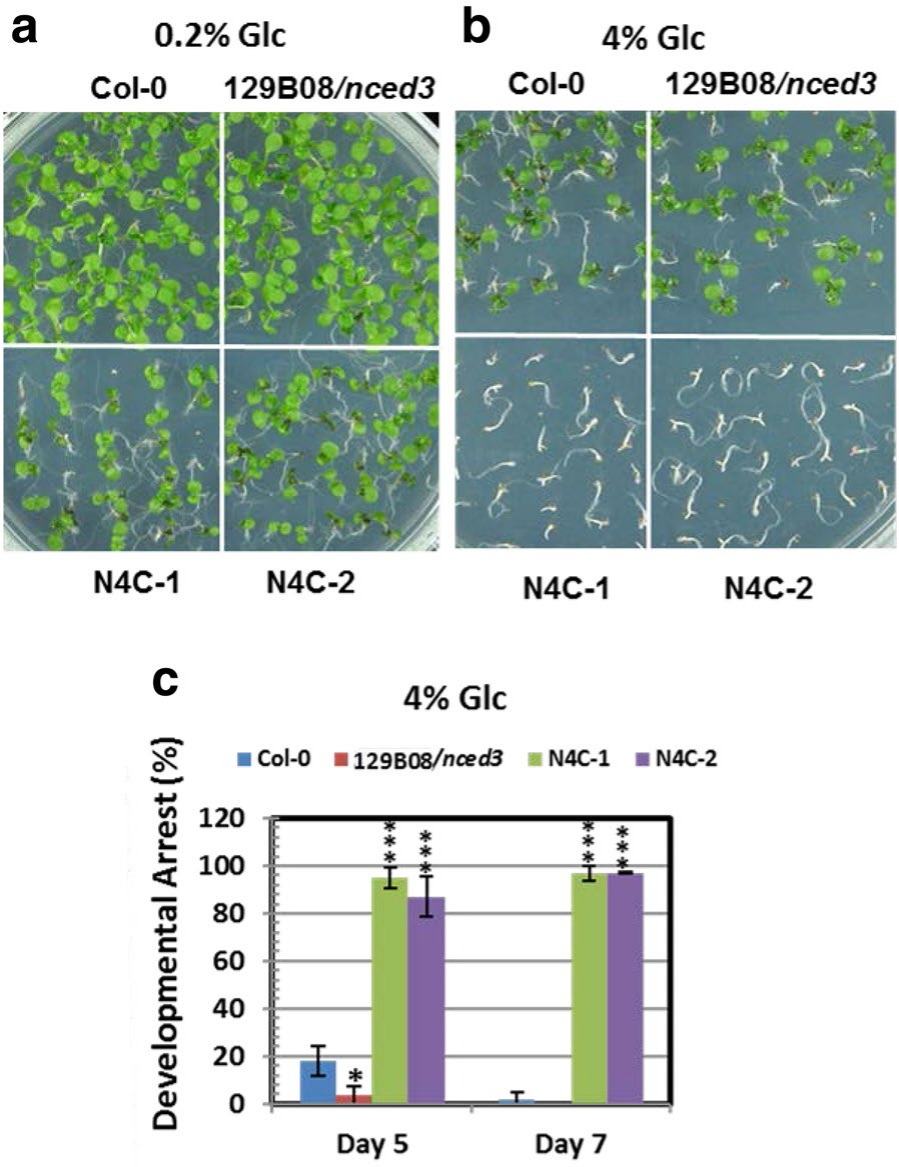
Heterologous expression of rice *OsNCED4* induced post-germination developmental arrest under high glucose condition. **a** Col-0, 129B08/*nced3* mutant, and N4C-1 and -2 transgenic seedlings grown on agar medium supplemented with 0.2% Glc for 7 days. **b** Col-0, 129B08/*nced3* mutant, and N4C-1 and -2 transgenic seedlings grown on agar medium supplemented with 4% Glc for 7 days. **c** The percentage of seedlings displaying post-germination developmental arrest at 5 and 7 days after plating on media containing 4% Glc. The values are the mean ± SD of three independent experiments, each with 80–100 seeds. *P < 0.05; ***P < 0.001, Student’s *t* test

After growing on agar medium containing 0.2% Glc for 7 days, the two N4C lines showed slower leaf growth compared to Col-0 and 129B08/*nced3* mutant plants (Fig. 4a). When grown on agar medium supplemented with 4% Glc for 7 days, the N4C transgenic lines showed post-germination developmental arrest, achieving neither cotyledon greening nor leaf formation (Fig. 4b). The percentage of N4C seedlings displaying post-germination developmental arrest was significantly higher than Col-0 and the 129B08/*nced3* mutant under high Glc conditions (4% Glc) (Fig. 4c; *P* < 0.001).

**Fig. 5:**
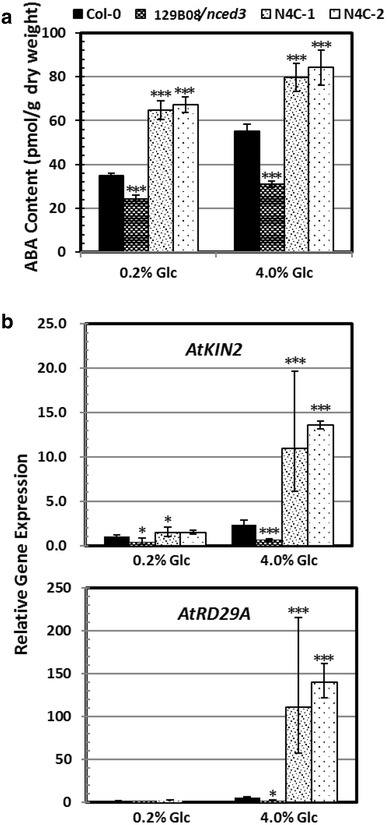
Transgenic Arabidopsis plants overexpressing *OsNCED4* had increased ABA content and expression of ABA-mediated stress-responsive genes. **a** ABA content in seedlings of Col-0, 129B08/*nced3* mutant, and two N4C transgenic lines grown on agar medium supplemented with 0.2 or 4% Glc for 10 days. **b** Relative transcript levels of *AtKIN2* (upper panel) and *AtRD29A* (lower panel) in seedlings of Col-0, 129B08/*nced3* mutant, and two N4C transgenic lines grown on agar medium supplemented with 0.2 or 4% Glc for 10 days. The values are the mean ± SD of three independent experiments. *P < 0.05; ***P < 0.001, Student’s *t* test

The ABA content was significantly lower in the 129B08/*nced3* mutant but significantly higher in the two N4C lines under both low and high Glc conditions compared to Col-0 (Fig. 5a). The transcripts of two ABA-regulated genes, *AtKIN2* and *AtRD29A*, varied in a pattern similar to the ABA content among the tested genotypes under both low and high Glc conditions (Fig. 5b), except that *AtRD29A* showed only minor signals in all lines under low Glc.

### Heterologous expression of *OsNCED4* in the 129B08/*nced3* mutant affected relative water loss and drought tolerance

**Fig. 6:**
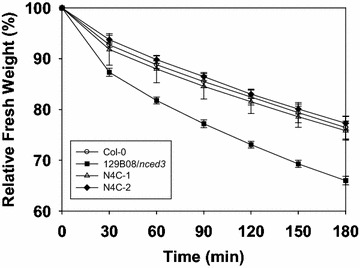
Relative fresh weight of rosette leaves excised from 3-week-old Col-0, 129B08/*nced3* mutant, and two N4C transgenic lines

**Fig. 7:**
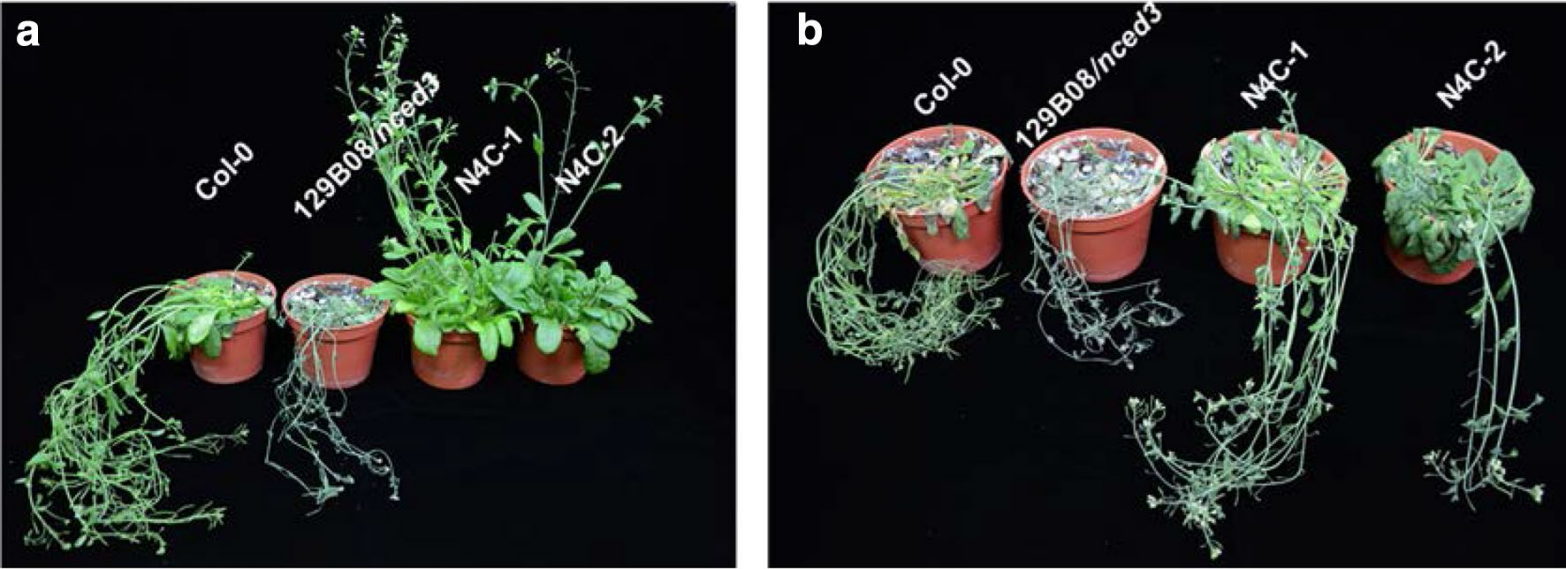
Overexpression of *OsNCED4* in transgenic Arabidopsis plants conferred drought tolerance. **a** Col-0, 129B08/*nced3* mutant, and two N4C transgenic lines after 9 days of withholding water. **b** Col-0, 129B08/*nced3* mutant, and two N4C transgenic lines after 13 days of withholding water

Relative water loss tests revealed that the 129B08/*nced3* mutant plants displayed a more severe water loss phenotype than the rest genotypes tested (Fig. 6). After 9 days of withholding water, wild type plants began to wilt, 129B08/*nced3* mutant plants showed severe wilt symptoms, and N4C transgenic plants remained healthy (Fig. 7a). It was not until 13 days of water withholding that two N4C lines tested displayed wilt symptoms (Fig. 7b).

### Characterization of transgenic rice plants carrying *OsNCED* promoter::*GUS* constructs

Promoter analysis using PlantCare (http://bioinformatics.psb.ugent.be/webtools/plantcare/html/) (Lescot et al. 2002) revealed that *OsNCED3* and *OsNCED4* share many common *cis*-elements such as ABRE, ARE, HSE, and MBS in their promoters within two Kb upstream of ATG start codon (Additional file 2). These data suggest that they might play an overlapping role in response to abiotic stress.

**Fig. 8:**
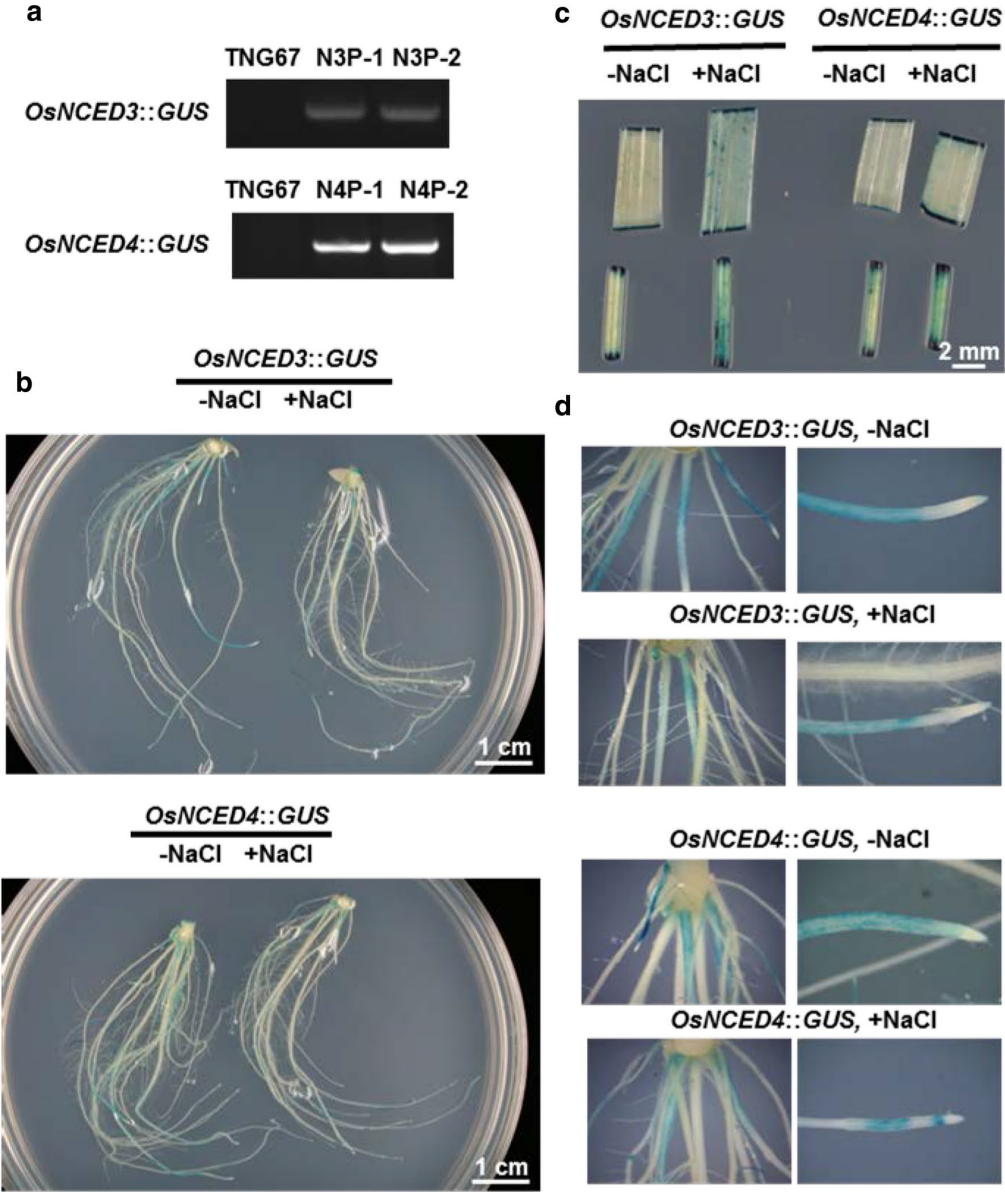
Comparison of tissue-specific expression of *OsNCED3* and *OsNCED4* promoters in transgenic rice plants under salt stress. **a** PCR analysis of *OsNCED3*::*GUS* or *OsNCED4*::*GUS* constructs in N3P or N4P transgenic rice plants, respectively. **b** GUS staining in roots of 7-day-old, solution-grown transgenic rice seedlings treated with (+ NaCl) or without (− NaCl) 150 mM NaCl. **c** GUS staining in leaf blade and leaf sheath of 7-day-old, transgenic rice seedlings grown in solution with or without 150 mM NaCl. **d** GUS staining in root segments of 7-day-old transgenic rice seedlings grown in solution with or without 150 mM NaCl

To understand and compare the expression patterns of *OsNCED3* and *OsNCED4* genes under both normal and stress conditions, transgenic rice plants carrying the *OsNCED3*::*GUS* or *OsNCED4*::*GUS* construct were generated. The presence of *OsNCED3*::*GUS* or *OsNCED4*::*GUS* constructs in the transgenic rice plants were verified by PCR. The forward primers were designed to bind the *OsNCED3* or *OsNCED4* promoter region and the reverse primer the GUS region. Amplicons of the *OsNCED3*::*GUS* and *OsNCED4*::*GUS* constructs were only detectable in homozygous N3P and N4P transgenic rice plants, respectively, but not in the TNG67 rice plant (Fig. 8a), indicating successful integration into the genomes of N3P and N4P transgenic rice plants.

### Expression analysis of *OsNCED3* and *OsNCED4* in rice seeds and seedlings

Tissue-specific expression analysis using RiceXPro database (http://ricexpro.dna.affrc.go.jp/) (Sato et al. 2011) indicated that both *OsNCED3* and *OsNCED4* genes are highly expressed during lemma and palea development (Additional file 3). As this prediction is based on bioinformatic analyses and the experimental data are still lacking. Thus, transgenic rice plants expressing *OsNCED3*::*GUS* and *OsNCED4*::*GUS* were used to confirm tissue-specific expression of *OsNCED3* and *OsNCED4*, respectively.

**Fig. 9:**
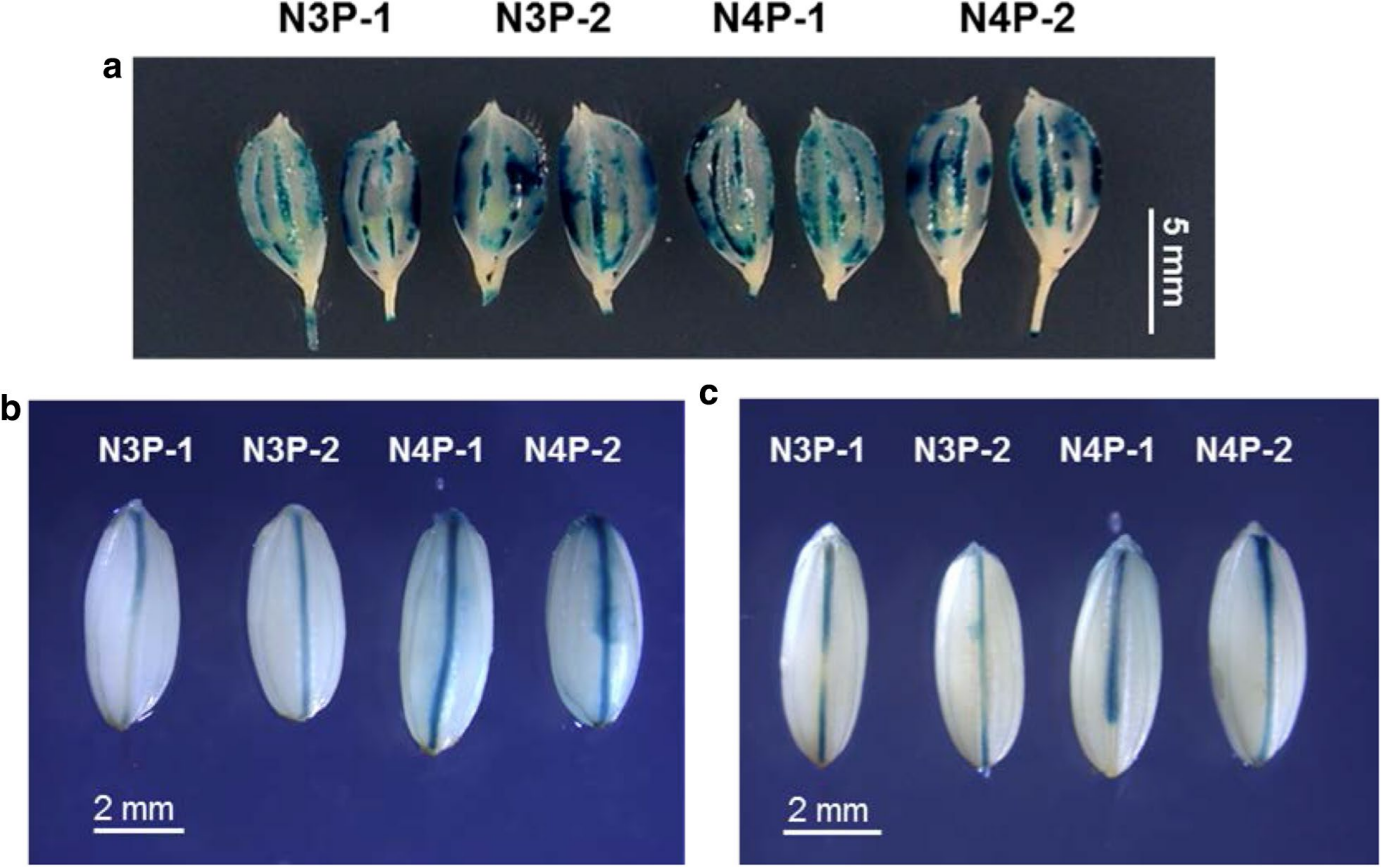
Tissue-specific expression of *OsNCED3* and *OsNCED4* in the husks and seeds of transgenic rice plants. **a** GUS staining in husks of *OsNCED3*::*GUS* (N3P) and *OsNCED4*::*GUS* (N4P) transgenic rice plants. **b** GUS staining in dry seeds of N3P and N4P transgenic rice plants. **c** GUS staining in imbibed seeds of N3P and N4P transgenic rice plants. In **a**, the husks were harvested at heading stage. In **c**, the seeds were imbibed in water at 25 °C for 24 h

Results from GUS staining revealed that both *OsNCED3* and *OsNCED4* were expressed in roots under both no-salt and salt-stress conditions, although the GUS signal appeared to be weaker in the salt-treated roots (Fig. 8b, d). In contrast, GUS signals in leaf blade and leaf sheath were enhanced by salt treatment (Fig. 8c). In N3P and N4P rice, whole grains harvested at heading stage showed strong GUS signals in the veins of the husks (Fig. 9a). In both dry (Fig. 9b) and imbibed (Fig. 9c) N3P and N4P seeds, GUS signals were observed in the aleurone layer. Interestingly, the GUS signals in N4P seeds were stronger than those in dry or imbibed N3P seeds (Fig. 9b, c).

## Discussion

Based on the NCBI GenBank database, there are more than three *NCED* genes in the rice genome. Welsch et al. (2008) investigated three rice *NCED* genes, namely *OsNCED3*, *OsNCED4*, and *OsNCED5*, and found that all three *NCED* genes are salt- and ABA-inducible. Previous research indicated that *OsNCED3* is functionally active in ABA biosynthesis, because its heterologous expression in Arabidopsis increases ABA levels (Hwang et al. 2010). The monocot *OsNCED3* gene was shown to alter leaf shape and regulate the development of vascular bundles in Arabidopsis (Hwang et al. 2010). However, it remains largely unknown if other rice *NCED* genes are involved in ABA biosynthesis and leaf morphogenesis.

NCBI blast analysis indicated that amino acid sequences of OsNCED3 and OsNCED4 have high levels of identity (Additional file 1). Additionally, results from promoter analysis of *OsNCED3* and *OsNCED4* within 2407 and 1939 bp upstream of the ATG start codon, respectively using PlantCARE indicated that both gene promoters contain multiple stress-responsive *cis*-acting elements (Additional file 2). Furthermore, tissue-specific expression analysis of *OsNCED3* and *OsNCED4* genes using RiceXPro database revealed that both genes are highly expressed during lemma and palea development (Additional file 3). Taken together, these results led us to investigate the potential function of *OsNCED4* in ABA biosynthesis.

We have previously transformed *35S*::*OsNCED3* into rice TNG67 and obtained some transgenic rice plants. Unfortunately, the transgene in these transgenic rice plants appeared to be leaky. In other words, the transgenic rice plants overexpressing *35S*::*OsNCED3* had transgene integration (based on antibiotic selection), but without transgene expression. It is most likely that the ABA balance is required during somatic embryogenesis of rice (a monocot crop) and disturbance of ABA balance will block somatic embryogenesis during rice transformation. To ensure accuracy of *35S*::*OsNCED3* molecular construct, *35S*::*OsNCED3* was heterologously transformed into Arabidopsis and the overexpression of *OsNCED3* was able to complement the ABA-deficient phenotypes in the 129B08/*nced3* mutant and increase ABA content in wild type (Hwang et al. 2010). Following the same reasoning, *35S*::*OsNCED4* transgene was overexpressed in Arabidopsis in this study. On the other hand, functional transgenic rice plants harboring *OsNCED3*::*GUS* or *OsNCED4*::*GUS* were obtained because not *NCED* coding sequence but *NCED* promoter sequence was present in the transgenic region and the ABA content and signaling pathway presumably were not changed in these transgenic rice plants.

### Heterologous expression of *OsNCED4* in the 129B08/*nced3* mutant delays seed germination and plays a role in shaping leaf morphology

To investigate the potential role of *OsNCED4* in ABA biosynthesis, a complementation test was performed using transgenic Arabidopsis plants carrying the *35S*::*OsNCED4* transgene in the 129B08/*nced3* mutant background. In the two complementation lines (N4C-1 and N4C-2), delays in seed germination were noticed under no, low- and high-Glc conditions (Figs. 2b, 3a, b). These results strongly suggested the possible involvement of *OsNCED4* in ABA biosynthesis, because ABA delays seed germination (Lin et al. 2007; Liu et al. 2016). A positive feedback regulation of ABA biosynthesis may cause the higher germination rate observed in the 129B08/*nced3* mutant relative to Col-0 under high Glc condition, a stress coping mechanism suggested by Xiong and Zhu (2003). Additionally, results from this research suggested that *OsNCED4* might share a redundant role with *OsNCED3* in regulating leaf morphology and ABA biosynthesis (Hwang et al. 2010), since heterologous expression of these two genes in Arabidopsis exerted similar effects on plant size, leaf shape and seed germination.

### Heterologous expression of *OsNCED4* in the 129B08/*nced3* mutant confers sugar oversensitivity

Post-germination growth was slightly reduced when the two *OsNCED4*-overexpressing lines were grown on medium containing low amounts of Glc, but it was severely inhibited under high Glc condition (Fig. 4a, b). These results were consistent with previous research indicating that Glc-induced physiological responses are different between low and high concentrations (Price et al. 2003). Interestingly, the percentage of seedlings that displayed post-germination developmental arrest was in accordance with seedling ABA content among all genotypes tested under high Glc conditions (Figs. 4c, 5a). In lines with this observation, the transcription levels of two ABA-regulated genes, *AtKIN2* and *AtRD29A*, varied with ABA content in the seedlings of the different genotypes exposed to high Glc (Fig. 5b). These results were in agreement with a previous finding suggesting that sugar-induced seedling growth arrest involves ABA biosynthesis and ABA signaling (Cheng et al. 2002; Dekkers et al. 2008). The sugar-oversensitive phenotype and the higher ABA content in the two *OsNCED4*-overexpressing lines together suggested a role for *OsNCED4* in ABA biosynthesis.

### Heterologous expression of *OsNCED4* complements the relative water loss and increases drought tolerance in the 129B08/*nced3* mutant

The roles of ABA in inhibiting stomatal opening and stimulating stomatal closure to achieve reduction in water loss are well documented in the literature (e.g., Kim et al. 2010; Sah et al. 2016). Results from relative water loss tests clearly showed that overexpression of *OsNCED4* in the 129B08/*nced3* mutant background successfully complements the severe water loss phenotype of the mutant to a level similar to that of Col-0 (Fig. 6). These results were consistent with the idea that *OsNCED4* is functionally active in ABA biosynthesis.

To determine the effects on drought tolerance of heterologous expression of *OsNCED4* in the 129B08/*nced3* mutant, 53-day-old plants (Col-0, 129B08/*nced3*, *OsNCED4*-*OE*) were left unwatered. After 9 or 13 days of withholding water, rosette leaves in the two *OsNCED4* complementation lines were less wilted than those of Col-0 or 129B08/*nced3* (Fig. 7a, b). These results indicated that heterologous expression of *OsNCED4* in 129B08/*nced3* not only complements the severe wilting phenotype of the mutant but also increases its drought tolerance to a level higher than that of Col-0. Results from this experiment further supported that *OsNCED4* is involved in ABA biosynthesis.

### Overlapping expression patterns of *OsNCED3*::*GUS* and *OsNCED4*::*GUS* reporter genes in rice seeds and seedlings

In this study, the GUS reporter gene was used to detect promoter activity of *OsNCED3* and *OsNCED4* under normal and salt-stress conditions. In 1-week-old transgenic rice seedlings at the one-leaf stage, overlapping GUS expression patterns were noticed. Interestingly, control roots (− NaCl) showed stronger GUS signal compared to those treated with 150 mM NaCl. This result was different from previous result documented by Welsch et al. (2008), where both *OsNCED3* and *OsNCED4* genes were found to be upregulated in 3-week-old seedling roots under salt stress (250 mM NaCl). This discrepancy may be explained by various seedling age (1- vs 3-weeks old), different levels of salt stress (150 mM vs 250 mM NaCl), and/or different lengths of salt treatment (7 days vs 1–3 h). Furthermore, stronger GUS signals were identified in newly developed adventitious roots, whereas older adventitious roots with root hairs showed much weaker signals (Fig. 8b, d). Opposite responses to salt were seen in the leaf sheath and blade, in which stronger GUS signals were detected in response to salt stress (Fig. 8c). Taken together, these results suggested that these two rice *OsNCED* genes are similarly regulated in roots or leaves at the one-leaf stage under long-term, moderate salt stress (150 mM NaCl). It is not clear why *OsNCED* genes are down-regulated in 1-week-old, solution-grown seedling roots in response to moderate salt stress, and the physiological meaning behind this requires further exploration.

Using *OsNCED3*::*gfp* transgenic plants, Bang et al. (2013) reported that *OsNCED3* promoter activity was not detected in grains and only barely detectable in flowers under normal growth conditions. Our GUS staining data indicated that promoter activity was clearly present in the husk at the heading stage and in dry and imbibed transgenic rice seeds under normal growth conditions (Fig. 9a–c). This discrepancy may be due to the more sensitive nature of GUS in detecting weak promoter activity compared to green fluorescent protein (Mantis and Tague 2000).

The revelation that both *OsNCED3* and *OsNCED4* are expressed in rice seeds in an overlapping pattern means that further assays are needed to understand how these genes work together to control ABA biosynthesis in rice seeds. Future studies on expression of *OsNCED3* and *OsNCED4* in dry and imbibed seeds will require using quantitative real-time PCR, seed ABA assays, and germination tests on wild-type and *OsNCED3* and *OsNCED4* knockout mutant seeds. It is interesting to note that *OsNCED1* and *OsNCED3*, but not *OsNCED4*, were suggested to be major players in controlling ABA biosynthesis in rice roots (Shi et al. 2015).

## Conclusions

Heterologous expression of rice *OsNCED4* in the 129B08/*nced3* Arabidopsis mutant background resulted in smaller and rounder leaves, delayed seed germination, post-germination sugar oversensitivity, increased ABA content, and enhanced tolerance to drought. These results indicated that in addition to *OsNCED3*, *OsNCED4* is also functionally active in ABA biosynthesis at least in Arabidopsis. Results from GUS staining with transgenic rice plants further revealed that *OsNCED3* and *OsNCED4* are expressed in seeds and one-leafed seedlings in an overlapping pattern and that both genes are downregulated in roots and upregulated in leaves in response to long-term, moderate salt stress. Our results demonstrated the possible functional redundancy between the *OsNCED3* and *OsNCED4* genes, although results from GUS staining alone are not sufficient to reach this conclusion. In light of different results in previous reports indicating upregulation of *NCED* genes in response to salt stress, the physiological importance of downregulation of *OsNCED* genes in roots of young seedlings under salt stress remains elusive and awaits further investigation. The fact that stronger GUS signals were detected in N4P seeds compared to N3P seeds suggested that *OsNCED4* may play a more prominent role in controlling ABA biosynthesis in rice seeds. Nonetheless, more research is required to confirm this hypothesis.

## Additional files

**Additional file 1.** Amino acid sequence alignment of OsNCED3, OsNCED4, and AtNCED3. Asterisks indicate fully conserved nucleotides and dots indicate strongly conserved residues. The plastid-targeting transit peptide is underlined, and four conserved histidines required for activity are marked by squares.

**Additional file 2.** Promoter analysis of *OsNCED3* and *OsNCED4* indicated that both promoter sequences contain multiple stress-related *cis*-elements.

**Additional file 3.** Tissue-specific expression of *OsNCED3* and *OsNCED4* predicted by using the rice expression profile database (RiceXPro).

## Abbreviations

ABA: abscisic acid
LOX: lipoxygenase
NCED: 9-*cis*-epoxycarotenoid dioxygenase
PCR: polymerase chain reaction
GUS: β-glucuronidase
RT-PCR: reverse-transcription polymerase chain reaction
Glc: glucose
qRT-PCR: quantitative real-time polymerase chain reaction
GFP: green fluorescent protein

## References

[CR1] Agustí J, Zapater M, Iglesias DJ, Cercós M, Tadeo FR, Talón M (2007). Differential expression of putative 9-*cis*-epoxycarotenoid dioxygenases and abscisic acid accumulation in water stressed vegetative and reproductive tissues of citrus. Plant Sci.

[CR2] Bang SW, Park SH, Jeong JS, Kim YS, Jung H, Ha SH, Kim JK (2013). Characterization of the stress-inducible *OsNCED3* promoter in different transgenic rice organs and over three homozygous generations. Planta.

[CR3] Bauer H, Ache P, Lautner S, Fromm J, Hartung W, Al-Rasheid KA, Sonnewald S, Sonnewald U, Kneitz S, Lachmann N, Mendel RR, Bittner F, Hetherington AM, Hedrich R (2013). The stomatal response to reduced relative humidity requires guard cell-autonomous ABA synthesis. Curr Biol.

[CR4] Chen YH, Shen HL, Hsu PJ, Hwang SG, Cheng WH (2014). *N*-acetylglucosamine-1-P uridylyltransferase 1 and 2 are required for gametogenesis and embryo development in *Arabidopsis thaliana*. Plant Cell Physiol.

[CR5] Cheng WH, Endo A, Zhou L, Penney J, Chen HC, Arroyo A, Leon P, Nambara E, Asami T, Seo M, Koshiba T, Sheen J (2002). A unique short-chain dehydrogenase/reductase in Arabidopsis glucose signaling and abscisic acid biosynthesis and functions. Plant Cell.

[CR6] Dekkers BJ, Schuurmans JA, Smeekens SC (2008). Interaction between sugar and abscisic acid signalling during early seedling development in Arabidopsis. Plant Mol Biol.

[CR7] Endo A, Sawada Y, Takahashi H, Okamoto M, Ikegami K, Koiwai H, Seo M, Toyomasu T, Mitsuhashi W, Shinozaki K, Nakazono M, Kamiya Y, Koshiba T, Nambara E (2008). Drought induction of Arabidopsis 9-*cis*-epoxycarotenoid dioxygenase occurs in vascular parenchyma cells. Plant Physiol.

[CR8] Gao G, Zhang S, Wang C, Yang X, Wang Y, Su X, Du J, Yang C (2011). Arabidopsis *CPR5* independently regulates seed germination and postgermination arrest of development through LOX pathway and ABA signaling. PLoS ONE.

[CR9] Hsu YT, Kao CH (2003). Role of abscisic acid in cadmium tolerance of rice (*Oryza sativa* L.) seedlings. Plant Cell Environ.

[CR10] Hsu YT, Kao CH (2008). Distinct roles of abscisic acid in rice seedlings during cadmium stress at high temperature. Bot Stud.

[CR11] Huo H, Dahal P, Kunusoth K, McCallum CM, Bradford KJ (2013). Expression of *9*-*cis*-*epoxycarotenoid dioxygenase4* is essential for thermoinhibition of lettuce seed germination but not for seed development or stress tolerance. Plant Cell.

[CR12] Hwang SG, Chen HC, Huang WY, Chu YC, Shii CT, Cheng WH (2010). Ectopic expression of rice *OsNCED3* in Arabidopsis increases ABA level and alters leaf morphology. Plant Sci.

[CR13] Iuchi S, Kobayashi M, Taji T, Naramoto M, Seki M, Kato T, Tabata S, Kakubari Y, Yamaguchi-Shinozaki K, Shinozaki K (2001). Regulation of drought tolerance by gene manipulation of 9-*cis*-epoxycarotenoid dioxygenase, a key enzyme in abscisic acid biosynthesis in Arabidopsis. Plant J.

[CR14] Jefferson RA, Kavanagh TA, Bevan MW (1987). GUS fusions: β-glucuronidase as a sensitive and versatile gene fusion marker in higher plants. EMBO J.

[CR15] Jiang W, Yu D (2009). *Arabidopsis WRKY2* transcription factor mediates seed germination and post germination arrest of development by abscisic acid. BMC Plant Biol.

[CR16] Kim T, Böhmer M, Hu H, Nishimura N, Schroeder JI (2010). Guard cell signal transduction network: advances in understanding abscisic acid, CO_2_, and Ca^2+^ signaling. Annu Rev Plant Biol.

[CR17] Lefebvre V, North H, Frey A, Sotta B, Seo M, Okamoto M, Nambara E, Marion-Poll A (2006). Functional analysis of Arabidopsis *NCED6* and *NCED9* genes indicates that ABA synthesized in the endosperm is involved in the induction of seed dormancy. Plant J.

[CR18] Lescot M, Déhais P, Thijs G, Marchal K, Moreau Y, Van de Peer Y, Rouzé P, Rombauts S (2002). PlantCARE, a database of plant *cis*-acting regulatory elements and a portal to tools for in silico analysis of promoter sequences. Nucleic Acids Res.

[CR19] Lin PC, Hwang SG, Endo A, Okamoto M, Koshiba T, Cheng WH (2007). Ectopic expression of *ABSCISIC ACID 2/GLUCOSE INSENSITIVE 1* in Arabidopsis promotes seed dormancy and stress tolerance. Plant Physiol.

[CR20] Liu X, Hu PW, Huang MK, Tang Y, Li Y, Li L, Hou XL (2016). The NF-YC-RGL2 module integrates GA and ABA signalling to regulate seed germination in *Arabidopsis*. Nat Commun.

[CR21] Mantis J, Tague BW (2000). Comparing the utility of β-glucuronidase and green fluorescent protein for detection of weak promoter activity in *Arabidopsis thaliana*. Plant Mol Biol Rep.

[CR22] Martínez-Andújar C, Ordiz MI, Huang Z, Nonogaki M, Beachy RN, Nonogaki H (2011). Induction of 9-*cis*-epoxycarotenoid dioxygenase in *Arabidopsis thaliana* seeds enhances seed dormancy. Proc Natl Acad Sci USA.

[CR23] Merilo E, Jalakas P, Kollist H, Brosche M (2015). The role of ABA recycling and transporter proteins in rapid stomatal responses to reduced air humidity, elevated CO_2_, and exogenous ABA. Mol Plant.

[CR24] Oliver SN, Dennis ES, Dolferus R (2007). ABA regulates apoplastic sugar transport and is a potential signal for cold-induced pollen sterility in rice. Plant Cell Physiol.

[CR25] Price J, Li TC, Kang SG, Na JK, Jang JC (2003). Mechanisms of glucose signaling during germination of Arabidopsis. Plant Physiol.

[CR26] Sah SK, Reddy KR, Li J (2016). Abscisic acid and abiotic stress tolerance in crop plants. Front Plant Sci.

[CR27] Sato Y, Antonio BA, Namiki N, Takehisa H, Minami H, Kamatsuki K, Sugimoto K, Shimizu Y, Hirochika H, Nagamura Y (2011). RiceXPro: a platform for monitoring gene expression in japonica rice grown under natural field conditions. Nucleic Acids Res.

[CR28] Shi L, Guo M, Ye N, Liu Y, Liu R, Xia Y, Cui S, Zhang J (2015). Reduced ABA accumulation in the root system is caused by ABA exudation in upland rice (*Oryza sativa* L. var. Gaoshan1) and this enhanced drought adaptation. Plant Cell Physiol.

[CR29] Sun L, Sun Y, Zhang M, Wang L, Ren J, Cui M, Wang Y, Ji K, Li P, Li Q, Chen P, Dai S, Duan C, Wu Y, Leng P (2012). Suppression of 9-*cis*-epoxycarotenoid dioxygenase, which encodes a key enzyme in abscisic acid biosynthesis, alters fruit texture in transgenic tomato. Plant Physiol.

[CR30] Tan BC, Joseph LM, Deng WT, Liu L, Li QB, Cline K, McCarty DR (2003). Molecular characterization of the *Arabidopsis* 9-*cis* epoxycarotenoid dioxygenase gene family. Plant J.

[CR31] Vishwakarma K, Upadhyay N, Kumar N, Yadav G, Singh J, Mishra RK, Kumar V, Verma R, Upadhyay RG, Pandey M, Sharma S (2017). Abscisic acid signaling and abiotic stress tolerance in plants: a review on current knowledge and future prospects. Front Plant Sci.

[CR32] Waterland N, Campbell CA, Finer JJ, Jones ML (2010). Abscisic acid application enhances drought stress tolerance in bedding plants. Hortsci.

[CR33] Welsch R, Wüst F, Bär C, Al-Babili S, Beyer P (2008). A third phytoene synthase is devoted to abiotic stress-induced abscisic acid formation in rice and defines functional diversification of phytoene synthase genes. Plant Physiol.

[CR34] Xiong L, Zhu JK (2003). Regulation of abscisic acid biosynthesis. Plant Physiol.

[CR35] Zhu G, Ye N, Zhang J (2009). Glucose induced delay of seed germination in rice is mediated by the suppression of ABA catabolism rather than an enhancement of ABA biosynthesis. Plant Cell Physiol.

[CR36] Zhu G, Liu Y, Ye N, Liu R, Zhang J (2011). Involvement of the abscisic acid catabolic gene *CYP707A2* in the glucose-induced delay in seed germination and post-germination growth of Arabidopsis. Physiol Plant.

[CR37] Zimmerli L, Hou BH, Tsai CH, Jakab G, Mauch-Mani B, Somerville S (2008). The xenobiotic β-aminobutyric acid enhances Arabidopsis thermotolerance. Plant J.

