# Supplementary figures and images for: Heterologous expression of rice 9-cis-epoxycarotenoid dioxygenase 4 (OsNCED4) in Arabidopsis confers sugar oversensitivity and drought tolerance
Source: Bot Stud. 2018 Jan 15;59:2. doi: 10.1186/s40529-018-0219-9 (PMC5768580; doi:10.1186/s40529-018-0219-9)

## Slide 1
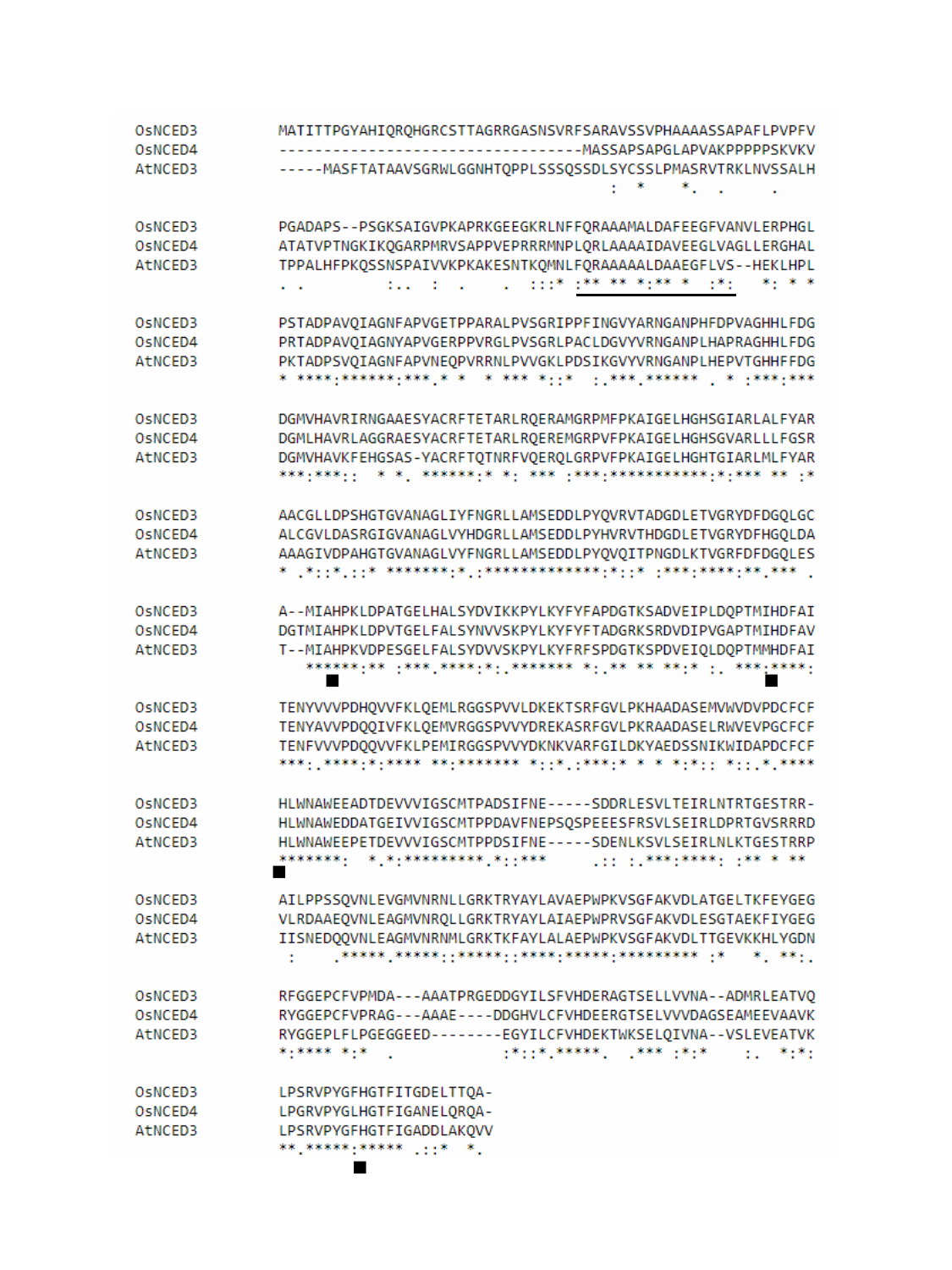

▪
▪
▪
▪

Supplement: Supplementary file 1 — Additional file 1. Amino acid sequence alignment of OsNCED3, OsNCED4, and AtNCED3. Asterisks indicate fully conserved nucleotides and dots indicate strongly conserved residues. The plastid-targeting transit peptide is underlined, and four conserved histidines required for activity are marked by squares. [file 40529_2018_219_MOESM1_ESM.pptx]

## Slide 1
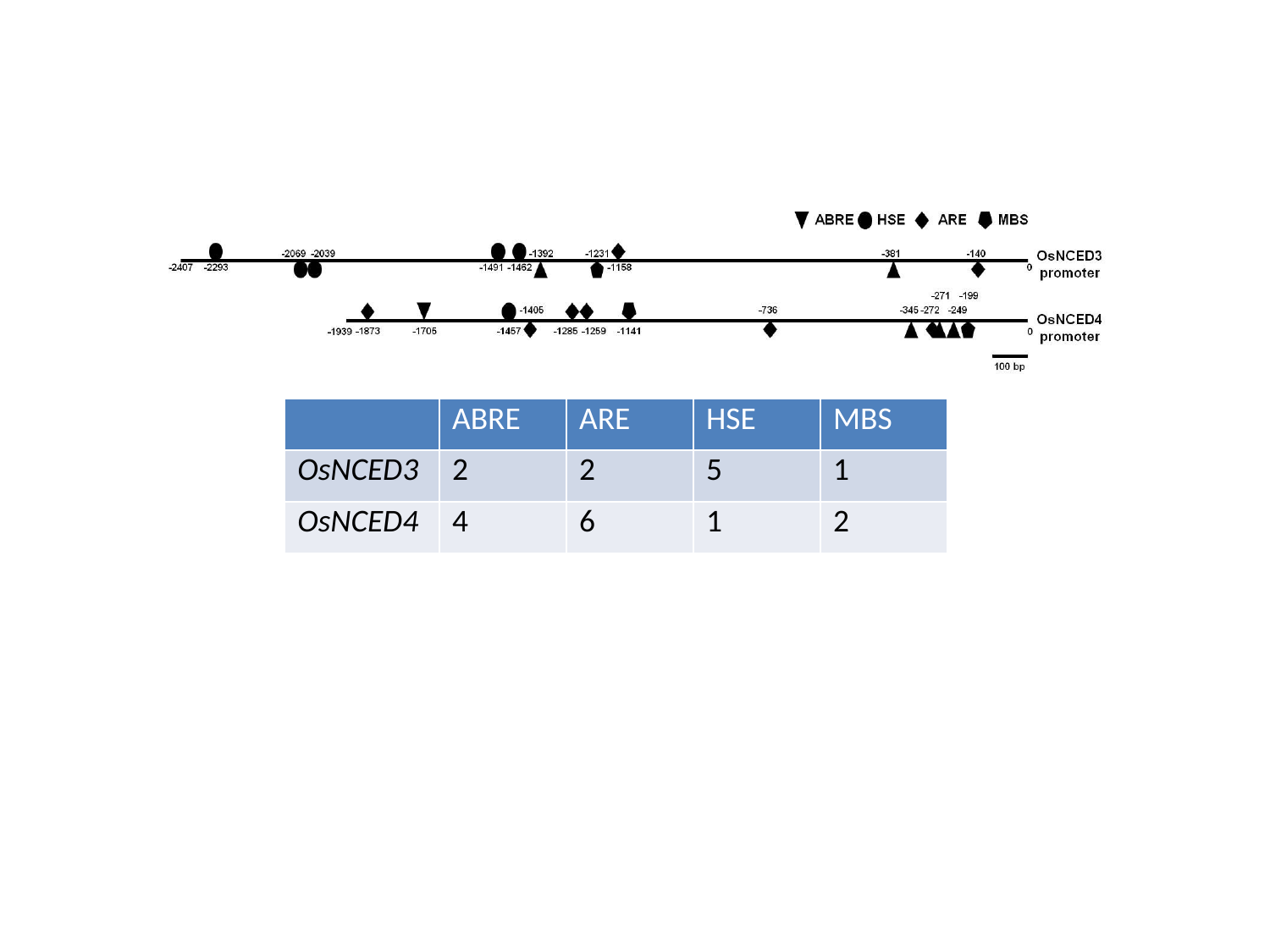

| | ABRE | ARE | HSE | MBS |
| --- | --- | --- | --- | --- |
| OsNCED3 | 2 | 2 | 5 | 1 |
| OsNCED4 | 4 | 6 | 1 | 2 |

Supplement: Supplementary file 2 — Additional file 2. Promoter analysis of OsNCED3 and OsNCED4 indicated that both promoter sequences contain multiple stress-related cis-elements. [file 40529_2018_219_MOESM2_ESM.pptx]

## Slide 1
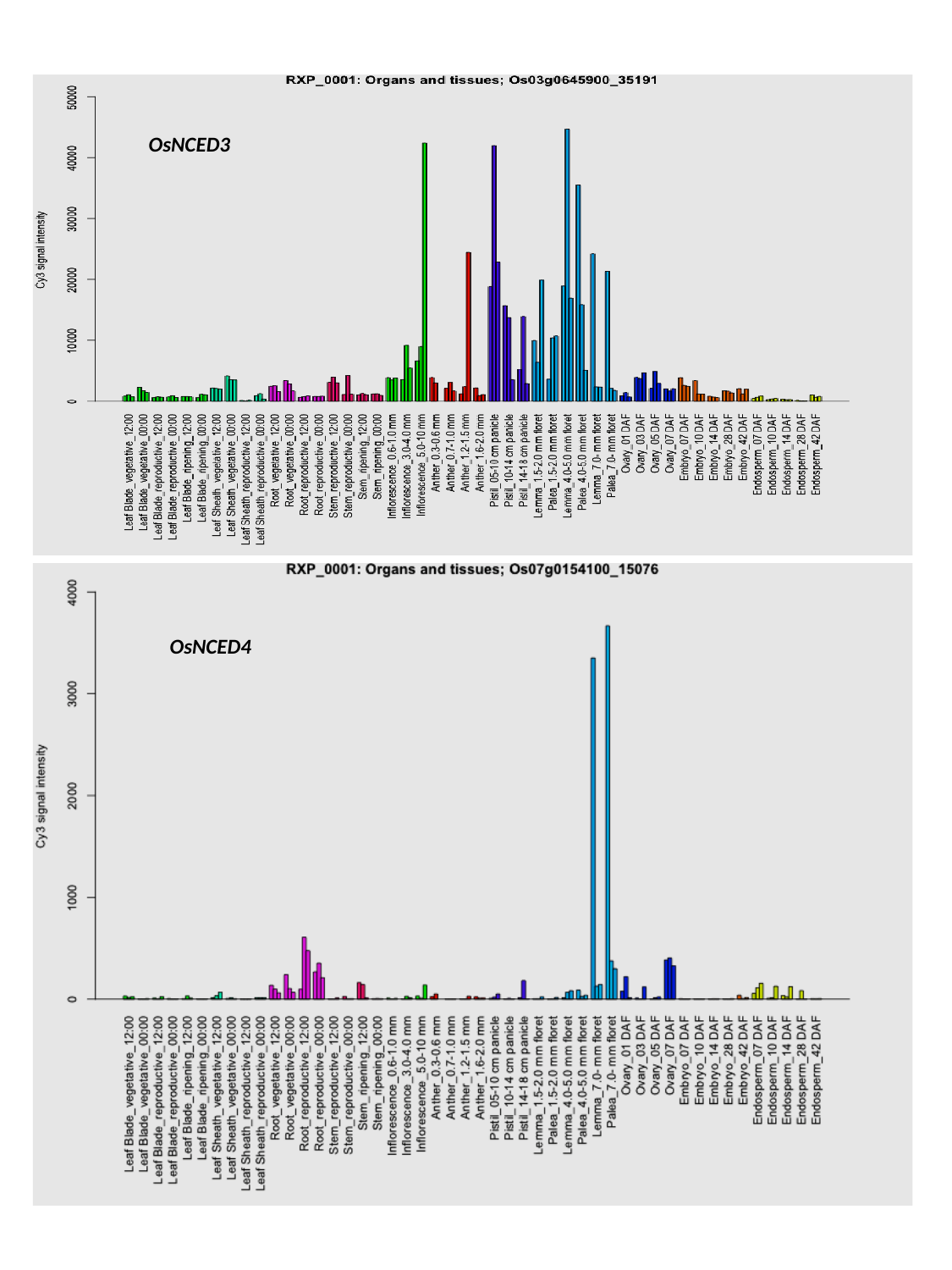

OsNCED3
OsNCED4

Supplement: Supplementary file 3 — Additional file 3. Tissue-specific expression of OsNCED3 and OsNCED4 predicted by using the rice expression profile database (RiceXPro). [file 40529_2018_219_MOESM3_ESM.pptx]
